# The Aqueous Soluble Polyphenolic Fraction of *Psidium guajava* Leaves Exhibits Potent Anti-Angiogenesis and Anti-Migration Actions on DU145 Cells

**DOI:** 10.1093/ecam/neq005

**Published:** 2011-06-08

**Authors:** Chiung-Chi Peng, Chiung-Huei Peng, Kuan-Chou Chen, Chiu-Lan Hsieh, Robert Y. Peng

**Affiliations:** ^1^School of Physical Therapy, College of Health Care, China Medical University, 91, Hsueh-Shih Rd., Taichung, Taiwan 40202, Taiwan; ^2^Graduate Institute of Rehabilitation Science, College of Health Care, China Medical University, 91, Hsueh-Shih Rd., Taichung, Taiwan 40202, Taiwan; ^3^Department of Nursing, Hungkuang University, 34, Chung-Chi Rd., Shalu County, Taichung Hsien, Taiwan 43302, Taiwan; ^4^Research Institute of Biotechnology, Hungkuang University, 34, Chung-Chi Rd., Shalu County, Taichung Hsien, Taiwan 43302, Taiwan; ^5^Department of Urology, Taipei Medical University-Shuang Ho Hospital, Taipei Medical University, 250, Wu-Xin St., Xin-Yi District, Taipei, Taiwan; ^6^Graduate Institute of Biotechnology, National Chang-Hua University of Education, 1, Jin-De Road, Changhua City 500, Taiwan

## Abstract

The aqueous extract of *Psidium guajava* budding leaves (PE) bears an extremely high content of polyphenolic and isoflavonoids. Whether it could be used as an anti-tumor chemopreventive in view of anti-angiogenesis and anti-migration, we performed the assay methods including the MTT assay to examine the cell viability; the ELISA assay to test the expressions of VEGF, IL-6 and IL-8; the western blot analysis to detect TIMP-2; the gelatinolytic zymography to follow the expression of MMPs; the wound scratch assay to examine the migration capability; and the chicken chorioallantoic membrane assay to detect the suppressive angiogenesis. Results indicated that the IC50 of PE for DU145 cells was *∼*0.57 mg ml^−1^. In addition, PE effectively inhibited the expressions of VEGF, IL-6 and IL-8 cytokines, and MMP-2 and MMP-9, and simultaneously activated TIMP-2 and suppressed the cell migration and the angiogenesis. Conclusively, PE potentially possesses a strong anti-DU145 effect. Thus, clinically it owns the potential to be used as an effective adjuvant anti-cancer chemopreventive.

## 1. Introduction

Angiogenesis is a highly regulated process essential to reproduction and wound healing. Uncontrolled angiogenesis could induce a number of diseases, including arthritis, diabetes-related blindness, psoriasis, tumor growth and metastasis. As well known, hypoxia is closely related with many common features of solid tumors [1] and is the most potent stimuli for expression of the vascular endothelial growth factor (VEGF) mediated primarily by the hypoxia inducible factor-1 (HIF-1), a master regulator of angiogenesis in hypoxia [2]. Pharmacologically, anti-angiogenic compounds are useful in treating diseases having uncontrolled angiogenesis, in contrast, pro-angiogenic compounds are beneficial to wound healing in minimizing tissue damages following ischemia injury. Literally, the angiogenesis induction during tumor growth and metastasis has become one of the focci in cancer research. Many natural products having multiple biological mechanisms are cumulatively considered to have chemopreventive effects [3, 4]. Accordingly, the anti-angiogenicity has become an alternative mechanism for identification of chemopreventive agents. Based on this, a diversity of assay methods has been developed to investigate both the pro- and anti-angiogenic effects of potential new drugs [5]. Literature elsewhere indicated that the activity of matrix metalloproteinases may be related with angiogenesis. However, only the MMP-13 (chMMP-13) in chicken embryo has been identified to be closely associated with its vascularization. Biochemically, chicken MMP-13 (chMMP-13) is the only enzyme whose induction and expression coincided with the angiogenesis and blood vessel formation [6]. In addition, many smaller peptides have been shown to bear more potent anti-angiogenic and inhibitory activities on the endothelial cell proliferation. Usually, these peptides are administered as one of the pharmaceutical compositions in the prevention and treatment of undesired angiogenesis, especially for the prevention of primary tumor growth and the inhibition of tumor metastasis (United States Patent 7317003).

*Psidium guajava* L. belongs to the family of Myrtaceae-Myrtle and the genus of *Psidium guajava* L.—guava [7]. *Psidium guajava* L. is an important tropical fruit widely grown in Taiwan, Hawaii, Thailand, Philippines and Malaysia. All parts of which including the fruits, leaves and barks have been traditionally used as the folkloric herbal medicines and exhibit many therapeutic uses including amebicide, analgesic, vermifuge, anti-malarial, anti-bacterial, colic-relief, anti-spasmodic, astringent, anti-ulcerous, gastrototonic cough suppressant, hypotensive, anti-inflammatory, diarrhea, some psychic diseases and hyperglycemia. Other documented medicinal uses are antianxiety, anti-spasmodic, anti-convulsant, antiseptic, blood cleanser, digestive and menstrual stimulants, infantile rotavirus enteritis, antiseptic, anti-oxidant, cardiodepressant, cardiotonic, central nervous system depressant, febrifuge and a topical remedy for ear and eye infections [7]. The aqueous extract of *P. guajava* L. (guava) budding leaf extract (PE) was reported to possess anti-oxidative, anti-glycative, anti-angiogenic effects [8], and anti-carcinogenic bioactivities [9], effects having been attributed to its extraordinary free radical scavenging and anti-oxidative capabilities. The high polyphenolic and flavonoid contents in PE are relevantly associated with its potent anti-glycative activity [10], implicating its beneficial effect for treatment of many cardiovascular and neural degenerative diseases [7]. More recently, we reported that PE contained significant amount of *β*-sitosterol glucoside and brahmic acid [7], apparently contributing to the lowering of plasma glucose levels in both non-diabetic and diabetic rats [7]. In this study, we try to elucidate the bioactivities of PE as anti-angiogenesis or anti-metastasis of prostate cancer using DU145 cells.

## 2. Materials and Methods

### 2.1. Reagent, Chemicals and Antibodies

Dulbecco's Modified Eagle Medium (DMEM), penicillin, streptomycin, fetal bovine serum (FBS), trypsin-EDTA and phosphate buffered saline (PBS) were purchased from Gibco (Langley, OK, USA). Acrylamide and the protein assay kit were obtained from Bio-Rad (Hercules, CA, USA). Polyclonal anti-MMP-2 and anti-MMP-9 were purchased from Sigma Chemicals (St Louis, MO, USA). TIMP-2 antibodies were obtained from Abcam (Taipei, Taiwan). *β*-Actin antibody was obtained from Chemicon (USA). All other chemicals used in this study were purchased from authentic sources and of highest grade and purity.

### 2.2. Preparation of Aqueous Extract of PE

PE was prepared according to the method previously described by Hsieh et al. [10].

### 2.3. Measurement of PE Components

#### 2.3.1. Water-Soluble Polysaccharide and Soluble Small Molecular Polyphenolics

The water-soluble polysaccharide fraction was isolated by similar method previously reported [11] with some modifications. Briefly, to 5.00 g of PE, 250 ml of deionized water was added. The mixture was boiled for 1 h and centrifuged at 3000 r.p.m. and for 30 min the sediment (2.31 g) was dried under nitrogen blowing to yield a residue of 0.24 g (SDI: recovery 4.80%). The supernatant (SI Super: 197.2 ml) was added with ethanol (95% v/v, 592 ml) to adjust the ethanol content to a final value of 71.25%. The mixture was agitated for 30 min and centrifuged at 10 000 g for 30 min. The sediment (SDII: wet weight 14.36 g) was lyophilized to give the water soluble polysaccharide fraction (SDIII *P*_ws_: dried weight 1.14 g, recovery 22.80%). The supernatant containing small molecular polyphenolics (SII Super, 752.04 ml) was evaporated under reduced pressure and then decanted out of the evaporation flask (the decant, the original concentrate). The empty flask was rinsed with the deionized water (dw, 100 ml). The washings were combined by decanting and subjected to lyophilization. The yield was 3.36 g based on the desiccated PE. This product in fact represented the small molecular polyphenolic fraction of PE (Φ_sm_: 3.36 g, recovery 67.2%).

#### 2.3.2. The Polysaccharides

To 0.5 mL of ADP, 0.5 mL of phenol color reagent (5%) was added. The mixture was vigorously agitated to facilitate a homogeneous solution. Sulfuric acid (2.5 ml) was rapidly dropped in. The mixture was agitated thoroughly to facilitate the color reaction (orange color). Optical density was measured at 490 nm.

#### 2.3.3. Total Polyphenolics

The desiccated herbal aqueous extract (5 mg) was dissolved in an acidic mixed solvent of methanol/water (60 : 40, 0.3% HCl). The following procedures were conducted according to Hsieh et al. [10]. The amount of polyphenolics present in the extracts was expressed as the gallic acid equivalents per gram sample, GAE/g.

#### 2.3.4. Total Flavonoids

The method described by Hsieh et al. [10] was followed. Briefly, to 1 mL of the aqueous solution of herbal extract (1 mg mL^−1^), 1.25 mL of deionized water was added and mixed thoroughly with 75 *μ*L of NaNO_2_ (5%). The remaining procedures were conducted in the same manner as described. The absorbance was measured at 510 nm and calculated against the calibration curve established using catechin as the reference standard and expressed as the catechin equivalents per gram sample, CE/g.

### 2.4. Cell Line and Cell Culture

Human prostate carcinoma DU-145 cells were purchased from the Culture Collection and Research Center (CCRC) of the Food Industry Research and Development Institute (FIRDI) (Hsinchu, Taiwan). DU-145 cells were cultured in RPMI 1640 media with 5% FBS and 1% penicillin-streptomycin cocktail (Cellgro, Mediatech, Inc., Herndon, VA, USA) at 37°C in a humidified atmosphere containing 5% CO_2_.

### 2.5. Cell Viability Assay

MTT assay was performed mainly by following the method described by Mosmann [12], yet modified by the manufacturer (Bio-Tek Instruments, VT, USA).

### 2.6. ELISA Assay for VEGF, IL-6 and IL-8

DU145 cells were seeded onto 24-well plates at a cell count of 2 × 10^4^ cells/well in a regular culture medium and incubated at 37°C for 48 h in the absence and the presence of PE at concentrations as indicated. The conditioned medium was collected and immediately frozen to −20°C for further use. Otherwise, these culture media were assayed fresh with a sandwich ELISA for VEGF, IL-6 and IL-8 according to the manufacturer's protocol (RayBiotech, Inc., Norcross, GA, USA).

### 2.7. Western Blotting Analysis

After incubated in the presence and the absence of PE, the DU145 cells were rinsed and lyzed following the standard procedure of cell preparation for Western Blotting [9]. The total protein extraction and quantification were performed as directed (BCA protein assay, Pierce, USA). The blotted membranes were incubated with TIMP-2 antibody (Abcam, Taipei, Taiwan). The immunoblotting signals were detected by chemiluminescence method as instructed (The ECL Western Blotting System, Amersham Pharmacia Biotech). The amount of proteins in gel slabs was quantified using a densitometer (ImagePro Plus 5.0 Medica Cybernetics). *β*-Actin was used as the internal control and treated with the same protocol [9].

### 2.8. Gelatinolytic Zymography

Gelatinolytic zymography was used to detect the expression of MMPs in supernatant media in presence or absence of PE as described by Leber and Balkwill [13] with slight modification. Briefly, the collected media (10 *μ*l) after treatment were loaded onto 10% sodium dodecyl sulfate (SDS)-polyacrylamide gel (SDS–PAGE) copolymerized with 0.1% gelatin and subjected to electrophoresis at 100 V for 1.5 h. In order to remove SDS, the gel was rinsed twice for 30 min, each with 2.5% Triton X-100 solution, then rinsed with incubation buffer (0.05 M Tris-HCl buffer, pH 8.0 containing 5 mM CaCl_2_ plus 5 mM ZnCl_2_) and incubated at 37°C overnight. The gel was stained with PhastGel Blue R at ambient temperature for 2 h. Gelatinases in media were detected as unstained degraded zones of gelatin on the gel and were quantified using a densitometer (ImagePro Plus 5.0 Medica Cybernetics).

### 2.9. Wound Scratch Assay

DU-145 cells (3 × 10^4^ ml^−1^) were seeded into 6-well tissue culture dishes and cultured in medium containing 10% FBS to confluent cell monolayers, which were then carefully wounded using sterile P200 pipette tips and removed any cell debris with PBS [14]. The cells were treated with PE at various concentration levels as indicated for 24 h and its migration was photographed under a phase contrast microscope. The cell migration ability can be expressed by the relative migration capability originally derived Liu et al. [15] with slight modification in this article

(1) $$R_{m}=\frac{S_{t}}{S_{u}},$$

where *R*_*m*_ is the relative migration capability (dimensionless). *S*_*t*_ is the migration distance of drug-treated cells (mm), and *S*_*u*_ is the migration distance of untreated cells (mm). Similar experiments were repeated in triplicates.

### 2.10. Chicken Chorioallantoic Membrane Assay

Fertilized chick eggs are incubated at 37°C and a specific humidity of 60% for 3 days (Incubators and More, Adelaide, Australia). A rectangular window (1 × 1.5 cm) was made in the eggshell and the eggs were replaced in the incubator without rotation until day 9 when filter paper disks saturated with PE (5 mg 200 *μ*l per egg) were placed on the chorioallantoic membrane. The normal unmanipulated chorioallantoic membrane was used as controls. The incubation was continued for 2 days further. The developing vasculature was observed once daily under a stereomicroscope. The extent of agiogenesis (the neovascular zones) was evaluated on the chorioallantoic membrane photographed at ×5 magnification by a dissecting microscope (SZ-CTV Olympus Optical Co, Ltd, Tokyo, Japan) attached with a digital camera (Panasonic GP_KR222, Panasonic, Osaka, Japan).

### 2.11. Statistics

The values were expressed as means ± SE. The significance between the control and treated groups was determined by Student's *t*-test.

## 3. Results

### 3.1. Tumor Cell Viability Was Suppressed in a Dose-Responsive Manner

**Figure 1:**
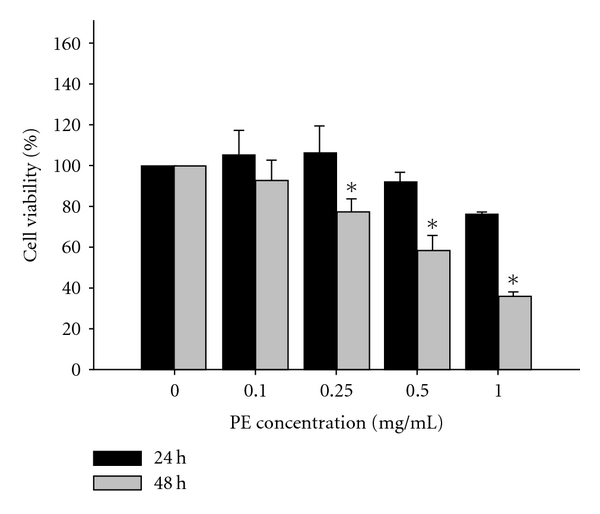
Effect of PE on the DU145 cell viability. The DU145 cells were exposed to PE at concentrations of 0.1, 0.25, 0.5 and 1.0 mg ml^−1^, respectively for 24 and 48 h. The viability (%) was determined by MTT assay. The vehicle-treated cells were used as the control (100%). Data were expressed in mean ± SD of the triplicates. *P < .05 to compare the cell viability between the treated and the untreated.

After incubation for 48 h PE suppressed the cell viability in a dose-responsive manner, from which the level of IC50 was estimated to be *∼*0.57 mg ml^−1^ (Figure 1).

### 3.2. Expression of VEGF Was Effectively Attenuated

**Figure 2:**
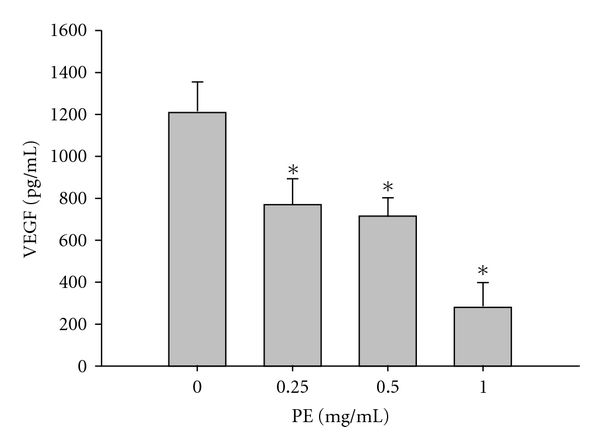
Effect of PE on VEGF expression in DU145 cells. DU145 was incubated at 37°C for 48 h in the absence or the presence of PE (0.25, 0.5, 1.0 mg ml^−1^). Data were expressed in mean ± SD of the triplicates. *P < .01 to compare the VEGF expression between the treated and the untreated.

The VEGF expression was effectively suppressed by PE at 0.25, 0.5 and 1.0 mg ml^−1^, the percent suppression attained 36.6, 41.2 and 76.91%, respectively (Figure 2) when compared with the control (1240 pg ml^−1^) taken as 100%. 

### 3.3. Anti-Angiogenesis Was Found by the Chicken Chorioallantoic Membrane Assay

**Figure 3:**
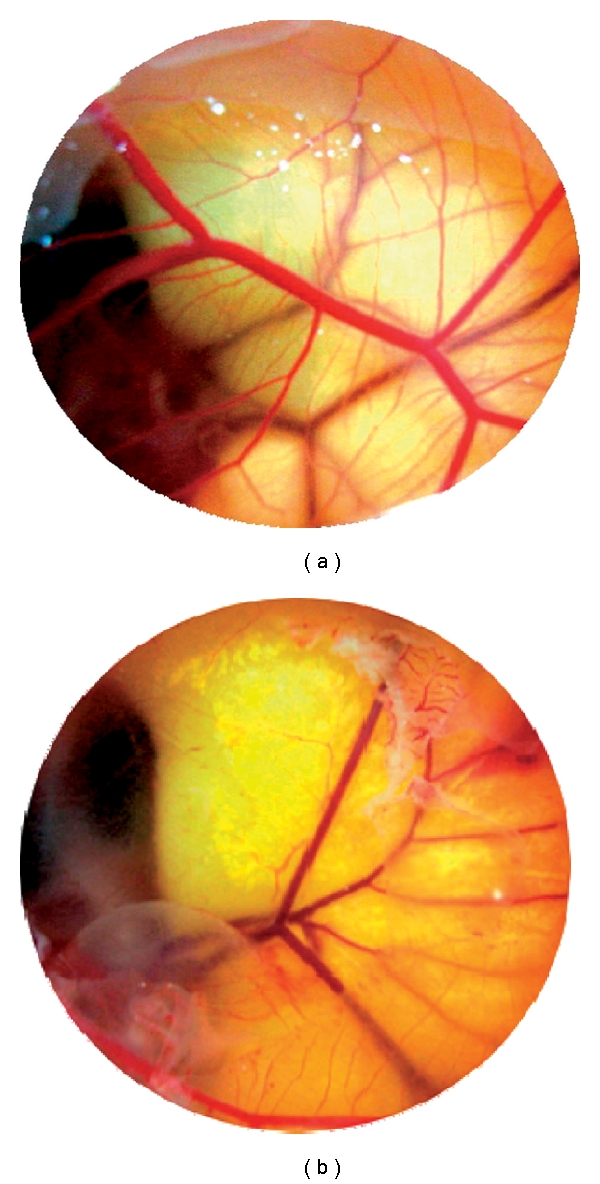
Effect of PE on angiogenesis. Chicken chorioallantoic membrane assay was conducted with addition of PE (200 *μ*l per egg of a solution of PE 25 mg ml^−1^) in a 9-day-old chicken embryo. The extent of neovascularization was examined 48 h after treatment in the absence (a) and the presence (b) of PE.

After the fertilized chicken egg received 200 *μ*l PE per egg (Figure 3) (concentration of PE, 25 mg ml^−1^), the angiogenesis was effectively suppressed (b) compared to the control (the untreated (a)). 

### 3.4. Expressions of IL-6 and IL-8 Were Prominently Suppressed

**Figure 4:**
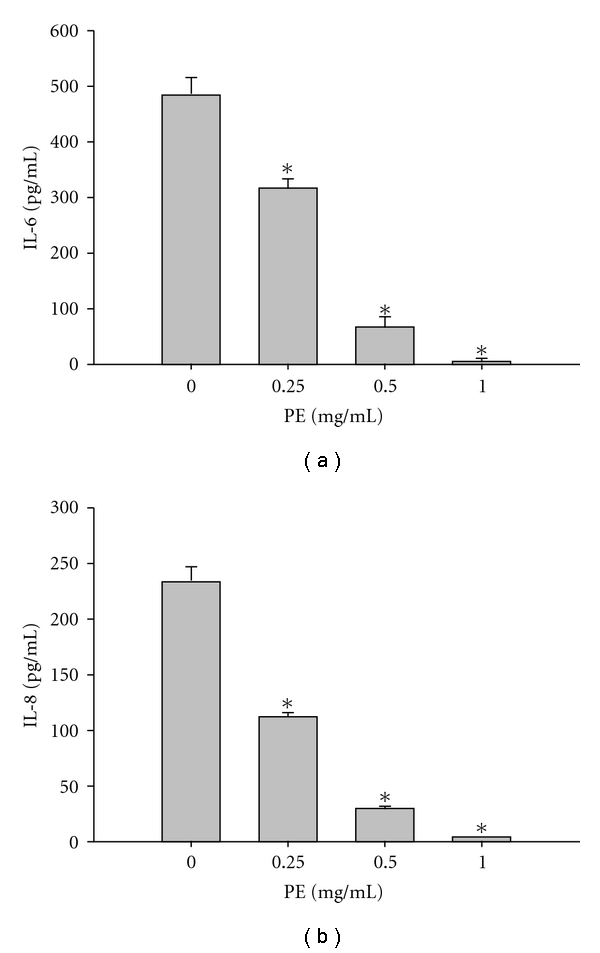
Effect of PE on expressions of IL-6 and IL-8 in DU145 cells. DU145 cells were incubated at 37°C for 48 h in the absence and the presence of PE at concentrations of 0.25, 0.5, 1.0 mg ml^−1^, respectively. Both IL-6 (a) and IL-8 (b) were effectively suppressed as shown. Data were expressed in mean ± SD of the triplicates. *P < .01 to compare the expressions of IL-6 and IL-8 between the treated and the untreated.

The expression of IL-6 also was apparently inhibited by PE at concentrations 0.25, 0.5 and 1.0 mg ml^−1^, respectively. The percent inhibitions obtained were 34.4, 86.0 and 98.8%, respectively (Figure 4(a)). Similarly, PE affected the expression of IL-8 as for IL-6 in DU145 cells. At indicated experimental concentrations, the percent inhibition was 51.6, 87.2 and 98.0%, respectively, at 0.25, 0.5 and 1.0 mg ml^−1^ (Figure 4(b)). 

### 3.5. PE Downregulated MMP-2, MMP-9 and Upregulated TIMP-2 in DU145 Cells

**Figure 5:**
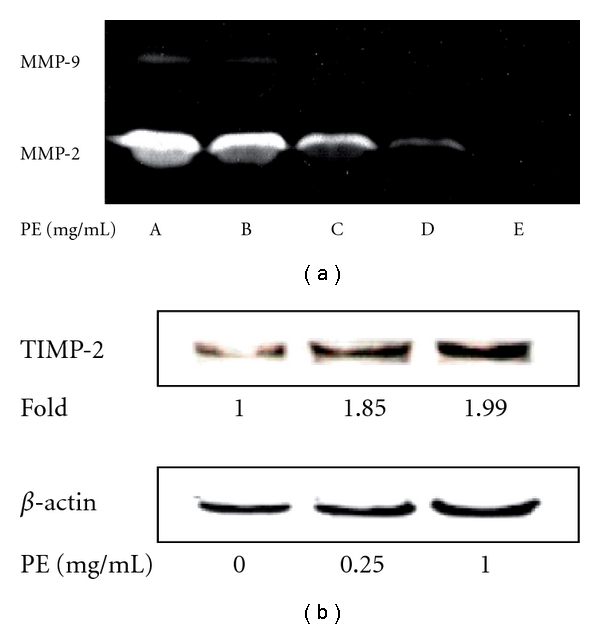
Effect of PE on expression of MMP-2 and MMP-9 in DU145 cells. DU145 cells (1 × 10^7^ cells) were treated with PE at concentrations (from A to E) 0, 0.1, 0.25, 0.5 and 1.0 mg ml^−1^ respective (A), and for TIMP2 assay: DU145 cells (1 × 10^7^ cells) were seeded onto a 10 cm dish in the absence or the presence of PE at concentrations of 0.25 and 1.0 mg ml^−1^, respectively. All cultures were incubated at 37°C for 48 h. The gelatin zymography showing the clear zones against the background reveals a dose responsive expression of both the matrix metalloproteinases MMP-2 and MMP-9 (a). For TIMP-2, an increased activity to 1.85- and 1.99-fold was indicated in the presence of 0.25 and 1.0 mg ml^−1^ of PE, respectively (b).

PE simultaneously downregulated both the matrix metalloproteinases, MMP-2 and MMP-9, in a dose-responsive manner. The MMP-9 was more susceptible to PE treatment than MMP-2. As can be seen, the complete inhibition of MMP-2 required a dose of PE 1.0 mg ml^−1^, whereas for MMP-9 only required a dosage of 0.25 mg ml^−1^ (Figure 5(a)). As for TIMP-2, an increased activity to 1.85- and 1.99-fold was indicated in the presence of 0.25 and 1.0 mg ml^−1^ of PE, respectively (Figure 5(b)). 

### 3.6. Tumor Cell Migration Was Effectively Inhibited by PE

**Figure 6:**
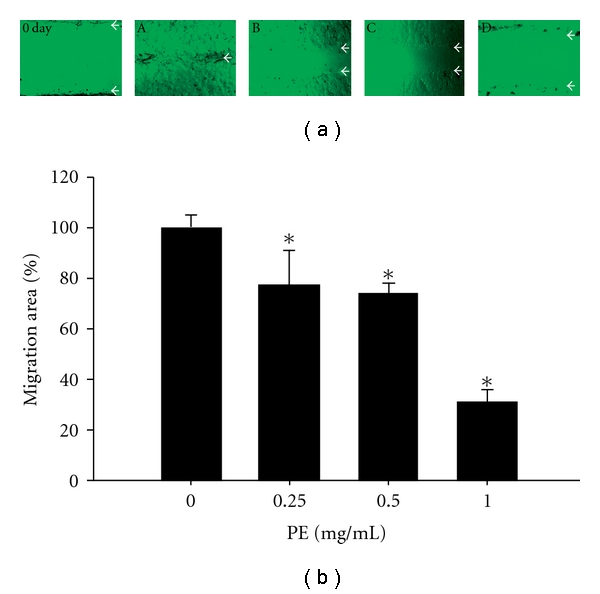
Effect of PE on the migration of DU145 cells. U145 cells (3 × 10^5^ cells/well) were seeded onto a 6-well plate. On 80% confluence, wound scratches were made with sterile P200 pipette tips. The plates were washed twice with PBS to remove detached cells. The cells were treated with PE at concentrations of A. 0, B. 0.25, C. 0.5 and D. 1 mg ml^−1^, respectively (a). After incubated for 24 h, the migrated distances were evaluated. Data were expressed in mean ± SD from triplicate experiments (b). *P < .05 compared with blank.

PE at concentrations as indicated effectively suppressed the cell migration of DU145 cells. The original wound scratch was as wide as shown in the upper left top picture indicated as 0 day. To low the PE dosages, the wounded area on the plate was completely healed (Figure 6(a), at PE 0.1 mg ml^−1^). When dosages of PE were increased, the cells became unable to across the wounded area of similar widths and failed to heal over the entire area. Instead, a gap was left behind as shown in B to D in a dose-responsive manner when incubated with PE 0.25, 0.5 and 1.0 mg ml^−1^, respectively (Figure 6(a)). Quantitatively, the migratory capability was correspondingly suppressed to 77.7, 74.0 and 30.9 %, respectively (Figure 6(b)). An implication is in the suppression of migratory capability of the DU145 cells by PE. 

## 4. Discussion

Often, expression of VEGF has been considered to be required for tumor cell proliferation and growth [1]. The binding of VEGF to VEGF receptor-2 (VEGFR-2) located on the endothelial cells brought forth the phosphorylation on VEGFR-2, which in turn activated the signaling pathways of Akt/PKB, p44/42MAP(ERK1/2) and p38MAPK [16–18]. As noted, the pathways Akt/PKB and ERK(1/2) are closely associated with the cell growth, proliferation and survival. In contrast, P38MAPK is related with the cell migration capability. Recently, Thejass and Kuttan [19] demonstrated that VEGF induced the migration and tubular formation in vascular endothelial cells. Similar phenomenon had also been confirmed in the prostate cancer LNCaP cells, the most popular type of prostate cancer.

Usually, IL-6 is expressed in the prostate stroma and basal epithelial cells. Frequently, it is highly expressed in some types of prostate cancers, which in turn participates in inducing angiogenesis in many cancer tissues [20]. Clinical data had shown highly expressed IL-6 and the prostate-specific antigen (PSA) in hormone-independent patients [20]. Under such a circumstance, IL-6 has been considered to act as the signaling messenger in many hormone refractory cancer cells, which in reality is associated with signaling pathway net including the mitogen-activated protein kinase (MAPK), the signal transducer and activators of transcription-3 (STAT-3), etc. As often cited, MAPK is related with cell growth and differentiation, while STAT-3 is able to activate the androgen receptor (AR) in the prostate cells through the expression of some neuropeptides. Obviously, to suppress the secretion of IL-6 will concomitantly inhibit the activation of AR. As a consequence, the progression of prostate cancer can be effectively retarded [21].

As a contrast, IL-8 usually is expressed in the normal prostate epithelium and DU145 cells. Its expression is closely related with the angiogenesis and the cell migration, which otherwise is interlinked with the upregulation of the extracellular-regulated kinase (ERK) [20]. In animal model, Oppenheim and Fujiwarat [22] indicated that the angiogenesis and cell growth were effectively suppressed when the IL-8 secretion was inhibited. As can be seen, PE efficiently suppressed the expression of IL-6 and IL-8, at dosage below 1.0 mg ml^−1^, implicating its future clinical applicability. The matrix metalloproteinases (MMPs) are frequently concerned with the tissue repairing and individual development. Although they were only slightly expressed, yet they were highly modulated. In tumor cells, they are always significantly expressed in the triggered cell migration. MMPs act on the extracellular matrix (ECM), resulting in the hydrolysis, degradation and separation of the intercellular ECM. Consequently, MMPs are closely related with the cancer migration and invasiveness. In addition, MMPs help the vascularization by hydrolyzing the ECM in blood vessels. Cell migration and angiogenesis are thus greatly enhanced resulting from the improved permeability and infiltration [22]. Among these both, the MMP-2 and MMP-9 contribute much more to the cancer invasiveness and metastasis [23]. In our experiment, PE downregulated the expression of MMP-2 and MMP-9, and activated TIMP-2, revealing a double strength anti-cancer effect.

**Figure 7:**
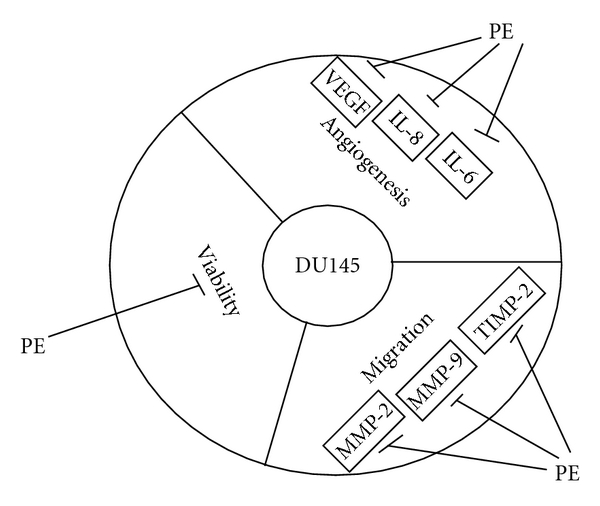
Action mechanism of the aqueous extract of PE on the DU145 cell line. The cell viability was prominently suppressed by PE. The angiogenesis was inhibited by PE through the downregulation of VEGF, IL-6 and IL-8. The cell migration was found inhibited through downregulation of MMP-2, MMP-9 and TIMP-2.

To summarize, the aqueous extract of PE leaves has shown amazing anti-cancer effects on the DU145 cells. The entire mechanism involved the suppression of cell viability, the reduced production of VEGF, the anti-angiogenic effect, the downregulation of IL-6 and IL-8, the inhibition on MMP-2 and MMP-9, and finally the activation of TIMP. The overall action mechanism of PE on DU-145 cells is shown in Figure 7.

Recent progress and promotion in the use of complementary adjuvant medicines (CAM) indeed has inspired the research interest and clinical trial of anti-cancer therapy. These reports can be of tremendous value in this regard.

Recently, our laboratory has identified that PE contained a tremendous polyphenolic compounds including gallic acid, catechin, epicatechin, rutin, quercetin, naringenin and kaempherol [8]. The total polyphenolic and isoflavonoid contents reached 229 and 208 mg g^−1^, respectively [24]. Previously, the anti-angiogenic capability of epigallocatechin-O-gallate (EGCG) and catechins has been demonstrated by Cao et al. [25], and Kang [5] indicated that EGCG inhibited angiogenesis in a dose-dependent fashion and reached an overall inhibition of 30–63% on the VEGF/vs3 signaling pathway. Literature also demonstrated that DNA fragmentation in DU145 cells was severely affected in the presence of 100–250 *μ*M of gallic acid, rutin, naringenin or kaempherol after an incubation period of 48 h [24]. Taken together, the polyphenolic and isoflavonoid compounds present in PE can contribute to its clinical uses.

## 5. Conclusion

The aqueous extract of PE bears an extremely high content of polyphenolic and isoflavonoids. The IC50 of PE to suppress the cell viability is *∼*0.57 mg ml^−1^. PE can effectively inhibit the expression of VEGF, IL-6 and IL-8 cytokines, and MMP-2 and MMP-9. In contrast, it activates TIMP and suppresses the cell migration and the angiogenesis. We conclude that PE potentially possesses a strong anti-prostate cancer effect. Results suggest that PE can be used as an effective clinical adjuvant anti-prostate cancer chemopreventive.
